# Supplementary material for: The Impact of a Multicomponent Platform Intervention on the Daily Lives of Older Adults
Source: Healthcare (Basel). 2023 Dec 5;11(24):3102. doi: 10.3390/healthcare11243102 (PMC10742799; doi:10.3390/healthcare11243102)
Supplement: Supplementary file 1 [file healthcare-11-03102-s001.zip › healthcare-2731516-supplementary.pdf]

Table S1. Baseline characteristics and test scores before the start of the experimentation (T0), at the midterm of the trial (T1) and at the end of the trial (T2) of the users

	User Code																								
	**U_01**	**U_02**	**U_03**	**U_04**	**U_05**	**U_06**	**U_07**	**U_08**	**U_09**	**U_10**	**U_11**	**U_12**	**U_13**	**U_14**	**U_15**	**U_16**	**U_17**	**U_18**	**U_19**	**U_20**	**U_21**	**U_22**	**U_23**	**U_24**	**U_25**
**Users' Characteristics**																									
Age	81	78	88	86	81	84	84	81	90	74	78	83	81	96	73	82	65	80	72	80	81	77	85	94	75
Female Gender	2	2	2	2	2	2	2	1	2	2	1	2	2	1	1	2	2	2	1	1	1	2	1	2	1
MMSE	21	27	26	25	24	22	23	27	26	24	27	25	26	27	27	26	27	27	27	27	29	24	27	24	26
FAC	5	5	3	5	5	5	5	5	5	4	5	5	5	5	5	4	4	5	5	5	5	5	5	4	5
Barthel	20	20	7	20	20	20	20	20	20	15	20	20	20	20	20	19	20	20	20	20		20	20	17	20
**T0**																									
SF-12 Physical Score	15	15	12	15	14	15	15	15	15	9	15	15	14	15	15	9	14	12	12	15	12	10	14	8	11
SF-12 Mental Score	16	20	20	18	15	17	17	19	17	17	16	17	21	20	18	19	19	19	18	17	20	20	15	17	11
Who-5	76	76	80	76	60	80	72	64	44	32	84	52	88	48	88	48	72	64	76	88	88	48	60	72	64
EQ-5D-5L	70	70	50	90	70	90	70	70	70	50	80	99	65	50	90	50	80	60	60	80	85	70	70	70	70
GSE Total Score	33	34	22	29	32	23	26	32	24	10	30	27	22	30	32	26	20	28	36	27	40	30	33	29	34
**T1**																									
SF-12 Physical Score	13	15	12	15	11	15	15	15	14	9	15	15	14	12	15	9	14	12	12	15	13	11	9	11	12
SF-12 Mental Score	15	20	20	17	15	17	17	17	14	17	16	18	20	19	18	19	18	19	18	17	18	18	18	22	15
Who-5	64	76	80	80	64	80	72	76	68	32	88	72	64	64	84	44	76	64	76	88	88	84	48	48	72
EQ-5D-5L	70	70	50	90	70	90	70	75	70	50	80	75	60	70	85	50	70	60	60	80	85	60	65	80	80
GSE Total Score	26	33	23	30	32	23	25	31	30	10	29	34	22	30	36	26	20	28	36	27	40	30	30	28	31
SUS Score	22.5	70	52.5	77.5	52.5	60	45	57.5	50	22.5	75	82.5	40	80	92.5	75	75	67.5	77.5	95	55	100	40	100	30
QUEST Score Watch	21	35	29	30	19	29	27	32	21	26	31	32	25	32	38	34	38	35	38	40	36	36	30	30	36
QUEST Score Kit	21	37	32	35	29	33	32	38	37	32	32	40	40	37	40	40	40	40	38	40	31				
**T2**																									
SF-12 Physical Score	15	14	12	15	10	15	15	15	14	9	15	15	14	13	15	11	14	12	12	15	14	9	10	12	10
SF-12 Mental Score	14	20	20	18	15	17	17	17	14	17	18	18	21	18	18	19	18	19	18	17	19	15	15	20	14
Who-5	60	76	80	76	64	80	80	76	68	32	84	72	52	64	84	52	76	64	76	88	80	60	60	48	84

EQ-5D-5L	70	70	50	90	70	90	75	80	70	50	85	80	60	70	90	60	80	60	60	80	80	70	100	80	80
GSE Total Score	30	34	23	30	25	24	26	31	30	10	32	34	22	30	32	27	20	28	36	27	39	33	34	28	38
SUS Score	27.5	72.5	52.5	77.5	45	50	50	57.5	50	22.5	70	82.5	40	67.5	92.5	82.5	75	67.5	77.5	95	60	95	90	50	82.5
QUEST Score Watch	28	35	29	31	26	27	27	32	26	26	31	32	25	32	38	34	38	35	38	40	38	39	33	34	38
QUEST Score Kit	29	37	32	38	32	34	34	38	36	32	32	40	40	40	40	40	40	40	38	40	35	35			26
